# Oxidative Stress-Dependent Coronary Endothelial Dysfunction in Obese Mice

**DOI:** 10.1371/journal.pone.0138609

**Published:** 2015-09-18

**Authors:** Ana María Gamez-Mendez, Hilda Vargas-Robles, Amelia Ríos, Bruno Escalante

**Affiliations:** 1 Departamento de Biomedicina Molecular, Centro de Investigación y de Estudios Avanzados (Cinvestav) del Instituto Politécnico Nacional, México, D.F. México; 2 Cinvestav Monterrey, Apodaca, N.L. México; University of Southampton, UNITED KINGDOM

## Abstract

Obesity is involved in several cardiovascular diseases including coronary artery disease and endothelial dysfunction. Endothelial Endothelium vasodilator and vasoconstrictor agonists play a key role in regulation of vascular tone. In this study, we evaluated coronary vascular response in an 8 weeks diet-induced obese C57BL/6 mice model. Coronary perfusion pressure in response to acetylcholine in isolated hearts from obese mice showed increased vasoconstriction and reduced vasodilation responses compared with control mice. Vascular nitric oxide assessed *in situ* with DAF-2 DA showed diminished levels in coronary arteries from obese mice in both basal and acetylcholine-stimulated conditions. Also, released prostacyclin was decreased in heart perfusates from obese mice, along with plasma tetrahydrobiopterin level and endothelium nitric oxide synthase dimer/monomer ratio. Obesity increased thromboxane A_2_ synthesis and oxidative stress evaluated by superoxide and peroxynitrite levels, compared with control mice. Obese mice treated with apocynin, a NADPH oxidase inhibitor, reversed all parameters to normal levels. These results suggest that after 8 weeks on a high-fat diet, the increase in oxidative stress lead to imbalance in vasoactive substances and consequently to endothelial dysfunction in coronary arteries.

## Introduction

Obesity is the result of an imbalance between calories consumed and calories expended. According to the World Health Organization (WHO) 65% of the world's population live in a country where overweight and obesity kill more people than underweight and this includes all high-income and most middle-income countries [1]. Obesity is a growing health problem that contributes to numerous life-threatening or disabling disorders, including coronary artery disease (CAD), hypertension, type 2 diabetes, and hyperlipidemia [2]. In addition, there is increasing evidence that obesity is a risk factor for endothelial dysfunction (ED). The endothelium regulates vascular tone through the synthesis and release of vasodilators and vasoconstrictors such as nitric oxide (NO), endothelium-derived hyperpolarizing factor (EDHF), prostaglandins (PGs), and thromboxanes [3, 4]. The term endothelial dysfunction has been used to refer to impairment in the endothelium capacity of keeping vascular homeostasis; this is the loss of antithrombotic and anti-inflammatory capacities. More specifically, ED has been defined as impairment of endothelium-dependent vasodilation caused by a loss of NO bioactivity in the vessel wall [5]. The association between obesity and ED has been widely described in humans and animal models. In a study conducted in humans, endothelium-dependent vasodilation was reduced by 40% in obese subjects with a body mass index ≥ 28 (BMI) compared with lean control subjects (BMI ≤ 28) under basal conditions [6]. Similarly, vasodilation response to acetylcholine (ACh) in arteries from Wistar rats fed with cafeteria diet showed significant reduced response compared with control rats [7]. Also, obesity has been strongly associated with increased synthesis of reactive oxygen species (ROS) that include superoxide and hydroxyl radicals, and reactive nitrogen species (RNS) formed by the reaction of superoxide with NO to produce peroxynitrite. These species are highly reactive molecules with unpaired electrons that quickly bind with nearby molecules; normal physiological processes require a carefully regulated production and availability of ROS and RNS. In excess, free radicals react with enzymes, nucleic acids, proteins, and carbohydrates modifying normal cell activities [8]. Roberts, *et al* (2000) found a decline in urinary nitrites and a significant raise in nitrotyrosine, a hallmark of NO inactivation by ROS, in aorta from rats fed for 24 months with a high-fat, refined carbohydrate diet [9]. Recently, we demonstrated in a diet induced obesity mouse model, that 2 weeks on a high fat diet are enough to increase superoxide anion levels in kidney tissue and to decrease urinary NO metabolites associated with a reduction in plasma tetrahydrobiopterin (BH4) concentration [10]. These changes were prevented by an antioxidant plus L-arginine treatment, suggesting that inactivation of endothelium nitric oxide synthase (eNOS) and the subsequent diminishment in NO bioavailability was related to the sequestration of NO by ROS and the uncoupling of eNOS by reduction in BH4 availability during obesity. Thus, obesity is intimately linked with ED through the presence of ROS and RNS along with reduction in NO production that leads to impaired vasorelaxation. The presence of this mechanism in coronary circulation may be associated with increased CAD. Therefore, we decided to evaluate the impact of obesity on the coronary vascular responses in a diet-induced obesity mice model and to characterize the role of ROS/RNS on vasodilation impairment. We tested ACh-dependent coronary vascular response in isolated perfused hearts from control and obese mice and explored the changes in NO and PGs metabolism associated with obesity. Moreover, an antioxidant treatment was used to evaluate whether ROS/RNS could be responsible for the impairment in NO-PGs pathway in obese animals.

## Materials and Methods

### Experimental Design

Male C57BL/6 mice (5–6 weeks old) were obtained from the Experimental Animal Care Center from Centro de Investigación y Estudios Avanzados del Instituto Politécnico Nacional (Cinvestav-IPN), México. All the procedures conformed to the National Institutes of Health “Guide for the Care and Use of Laboratory Animals (1996) and were approved by the Institutional Ethics Review Committee for Animal Experimentation of Cinvestav-IPN. All surgeries were performed under isoflurane anesthesia with a room air ventilator (Ugo Basile, Stoelting Co., IL, USA), and all efforts were made to minimize animal suffering. Mice were acclimated for 1 week and randomly divided into three experimental groups: control group fed with standard diet (SD, n = 10), high-fat diet group (Research Diets, HFD, n = 10) and HFD plus antioxidant treatment (HDF+Tx, n = 10). The HFD groups were fed with a HFD carrying 58% energy by fat on caloric basis. SD or HFD was administered by 8 weeks. The antioxidant-treated HFD group was included to determine whether free radicals had an effect in the changes observed in HFD mice. Antioxidant treatment (Apocynin, 2.4 mg/L in drinking water, Sigma Chemical Co., USA) began along with the HFD and finished until the end of the experiments. All animals had free access to food and water.

### Obesity Parameters Measurement

Since obesity is defined as an excessive amount of body fat in relation to lean mass, the adiposity index (AI) was calculated as previously described [11]. Briefly, animals were anesthetized then sacrificed, and fat pads (epydidimal, retroperitoneal, and mesenteric) were dissected and weighed. Also, hearts were carefully dissected and weighed for heart weight/body weight ratio (HW/BW) determination.

For glucose, cholesterol, and triglycerides measurement, mice were fasted for 12 hours and a capillary blood sample was immediately placed on test strip devices for the corresponding quantification (AccuCheck Sensor and Accutrend GCT, Roche). Plasma leptin was measured using an enzyme immunoassay kit following the manufacturer´s instructions (Cayman Chem. Co. Michigan, USA). The absorbance was read at 450 nm.

Intracarotid blood pressure (BP) was measured as previously described [12]. After 8 weeks on the specific diet, mice were deeply anesthetized with isoflurane/O_2_ (1.5%, 100–200ml/min) and the left carotid artery was cannulated with a polyethylene catheter (I.D.011′ OD .024′, Clay Adams, Nutley, NJ, USA) connected to a solid-state pressure transducer (DUO.18 WPI, Aston, UK) coupled to a data acquisition system (Lab-Trax 4/24T, WPI). BP was continuously recorded during 15 min and analyzed with DataTrax software (WPI, Sarasota, FL, USA). At the end of this period animals were sacrificed and heart dissected as described below. Results were plotted as mean arterial pressure.

### Coronary Vascular Responses

The Langendorff isolated perfused heart system [13] was used to evaluate coronary vascular responses and to determine the coronary perfusion pressure (CPP) responsiveness to ACh (Sigma Chemical Co., St. Louis, MO.). Deeply anesthetized mice were injected with heparin (100 IU). Hearts were excised, placed directly into ice-cold Krebs-Henseleit bicarbonate solution (in mM: NaCl 117.8; KCl 6.0; CaCl_2_ 1.6; MgSO_4_ 1.2; KH_2_PO_4_ 1.2; NaHCO_3_ 24.2; glucose 11; EDTA 0.027), and equilibrated with 95% O_2_: 5% CO_2_ (pH 7.4). Under a stereoscopic microscope the aorta was localized and cannulated with a rounded 20- gauge needle. Rapidly, hearts were placed in a Langendorff system and retrograde perfused with the Krebs solution infused through a peristaltic pump (3 mL/min) at 37°C. Spontaneously beating hearts were stabilized for 30 min and CPP was continuously monitored via a pressure transducer (UFI, Morro Bay, CA. USA) attached to a channel data acquisition recorder (Lab-Trax™-4/24T, WPI) and a BP display unit (Stoelting, Co., IL. USA). In order to minimize differences in the vascular responses associated with isolation or instrumentation procedures, three experiments were performed every day using one heart from every experimental condition (SD, HDF, and HDF+Tx), the order of the heart was exchanged every day.

A coronary vascular dose-response curve was performed by administration of increasing doses of ACh (13, 41, 82, 164, and 493 μM) in a constant volume of 10μL bolus into the Langendorff system, distal to the aortic cannula by means of individual microsyringes (Hamilton Company, Reno, NV. USA). CPP was recorded and analyzed with DataTrax2 software. For the rest of experiments, 164 μM of ACh was chosen since this dose elicited a biphasic vascular response in SD coronary arteries.

After hearts were stabilized for 30 min and pressure values remained constant, an initial ACh stimulation was performed. Subsequently, CPP was allowed to return to baseline after 30 min washout. Then, coronary vascular response was assessed by administration of a second ACh bolus in the presence or absence of inhibitors. Either the arginine analogue NG-nitro L-arginine methyl ester (L-NAME, 1mM, Sigma Chemical Co., St. Louis, MO.) or indomethacin (3mM, Cayman Chem. Co. Michigan, USA) were added in the Krebs solution and hearts were perfused 30 min before the second ACh stimulation. Responses to ACh were recorded as mmHg and expressed as percentage change. Coronary constriction was calculated as $\left[\frac{response}{baseline}*100\right]$, while coronary relaxation was calculated as $\left[100-\left(\frac{response}{baseline}*100\right)\right]\;$. To evaluate the endothelium independent relaxation, sodium nitroprusside (SNP, 0.01 M, Sigma Chemical Co., St. Louis, MO.) was used in phenylephrine (PE, 0.08 M, Sigma Chemical Co., St. Louis, MO.) pre-contracted coronary arteries from SD and HFD mice.

### *In situ* Nitric Oxide Detection

To detect intracellular NO during ACh stimulation, the excised heart was washed and stabilized with Krebs solution at 37°C and then an ACh bolus (164 μM) was injected. Quickly after this, the heart was placed in Tissue-Tek® O.C.T. ™ compound and frozen in liquid nitrogen. Cryostat sections (8μm) from the frozen heart were incubated for 2h at room temperature with diacetylated 4, 5-diaminofluorescein-2 (DAF-2 DA, 10μM, Enzo Life Sciences Inc. Farmingdale, NY). This dye is intracellularly hydrolyzed by cytosolic esterases releasing DAF-2 that in the presence of NO is converted into a fluorescent triazole derivative (DAF-2T) [14]. Images were obtained using a laser scanning confocal imaging system (Leica TCS SP5, Mannheim, Germany) with excitation and emission wavelengths of 490 and 510–560 nm, respectively. Images were analyzed for each vessel with ImageJ software (NIH). Fluorescence and background were determined and subtracted, and the resulting value was normalized to the scanned surface area.

### *In Situ* Superoxide and Peroxynitrite Detection

Superoxide and peroxynitrite generation in coronary arteries were evaluated with dihydroethidium (DHE, 10μM, Molecular Probes, Grand Island, NY) and hydroxyphenyl fluorescein (HPF, 20μM, Molecular Probes, Grand Island, NY), respectively. Unfixed frozen heart sections (12 μm) from every condition were incubated for 1 hour in a light-protected humidified chamber at 37°C. Images were acquired at 400x magnification, using identical instrument settings, with a laser scanning confocal imaging system (Leica TCS SP5, Mannheim, Germany) and excitation and emission wavelengths of 510/615 nm for superoxide and 490/515 nm for peroxynitrite detection. Images were analyzed for each vessel with ImageJ software (NIH). Fluorescence and background were determined and subtracted, and the resulting value was normalized to the scanned surface area.

### Protein Expression

eNOS monomer and dimer were quantified by western blotting via low-temperature (LT)-SDS-PAGE in heart homogenates from every experimental condition, as described previously [15]. The LT process was used to identify eNOS dimers and monomers in the native state, as LT is known to prevent monomerization of eNOS dimers. Briefly, excised hearts were deep-frozen in liquid nitrogen. Frozen tissues were homogenized with lysis buffer (50 mM Tris—HCl pH = 7.4, 137 mM NaCl, 2 mM EDTA, 1% NP-40, 5% glycerol) containing a protease inhibitor cocktail (Complete,Roche Diagnostics, Indianapolis, IN). The homogenate was centrifuged at 10,000*g* at 4°C, and supernatant was collected. Protein was measured by the BCA method and 80 μg of protein were resolved in a 10% LT-SDS-PAGE. All gels and buffers were pre-equilibrated to 4°C before electrophoresis and the gel temperature was maintained below 15°C. eNOS and GAPDH (1:500 and 1:1000, Abcam Inc., USA) were detected by enhanced chemiluminescence (ECL, Amersham Biosciences, Freiburg, Germany) and analyzed with 1D image analysis software (Kodak, Rochester NY, USA).

### Tetra and Dihydrobiopterins Quantification

Plasma BH4 and dihydrobiopterin (BH2) were analyzed by capillary zone electrophoresis as previously described [12]. Briefly, plasma was deproteinized in methanol (1:1 v/v), centrifuged at 15,000 g for 15 min and filtered prior to analysis in a P/ACETM MDQ system (Beckman Coulter, Brea, CA. USA). Samples were diluted with 0.1 M sodium hydroxide (NaOH) and the system was preconditioned with a cycle of NaOH (1.0 M) followed by distilled water, and ending with running buffer (0.1 M Tris, 0.1 M boric acid, 2 mM EDTA, pH 8.75), 30 min each. The separation was carried out using 20 kV for 10 min at 445 nm. The concentration was determined using a biopterin standard curve (0–2500 pmol/mL).

### Thromboxane A_2_ and Prostacyclin Determination

The excised heart was stabilized for 30 min with Krebs solution at 37°C, and 3 mL of perfusate were collected throughout this period of basal perfusion pressure. After injection of an ACh bolus (164 μM), perfusate was collected during contraction phase for measurement of TXB_2_, the inactive metabolite of thromboxane A_2_ (TXA_2_) and during relaxation phase for measurement of 6-keto prostaglandin F_1α_, the stable hydrolyzed product of unstable prostacyclin (PGI_2_). These metabolites were assessed in perfusates from SD and HFD hearts by enzyme-linked immunosorbent assay (Cayman Chemical, Ann Arbor, MI) according to the manufacturer´s instructions.

### Statistical Analysis

We evaluated all our data using one way ANOVA to demonstrate statistically significant differences between the experimental groups, followed by a Tukey test for multiple comparisons. P value < 0.05 was considered statistically significant. Results are presented as mean ± SEM.

## Results

### Effect of HFD on Metabolic and Biochemical Parameters

HFD mice compared with SD mice showed ~ 50% increment in body weight, and 53% and 9% increments in triglycerides and cholesterol, respectively. Also, adiposity index was 5-fold higher and plasma leptin showed a 2-fold increase; arterial blood pressure augmented 30%. No differences were observed either in fasten glucose or HW/BW ratio (Table 1). In addition, we did not find evidences of cardiac hypertrophy in the HDF mice compared with SD mice. The heart weight/ femur length measurements obtained were 8.3± 0.5 mg/mm, 9.± 0.8 mg/mm and 8.9±0.3 mg/mm for SD, HFD, and HFD+Tx mice, respectively. Ventricle wall thickness results were 1.14±0.05 mm, 1.12±0.06 mm, and 1.10±0.04 for the same groups. Also, no differences were found in the force of contraction of isolated perfused hearts from SD, HDF, and HDF+TX (0.28± 0.05, 0.25± 0.03, and 0.26± 0.05 mg tension/100mg heart tissue, respectively).

**Table 1:** Metabolic and biochemical parameters in obese mice.

Diet	BW (g)	AI (%)	BG (mg/ml)	TG (mg/ml)	CH (mg/ml)	LP (ng/ml)	HW/BW	BP (mmHg)
**SD**	27.3±2	1.7± 0.2	146±10	112.2±4	159.8±3	333±128	4.7±0.2	75±3
**HFD**	40.4±3*	9.5±0.4*	159±12	172.4±9*	174.4±2*	713±85*	4.8±.03	98±2*
**HFD+Tx**	32.8±1**	7.9±0.5**	90.6±8**	120±6**	164±3	204±48**	5.0±0.6	92±3

Standard diet (SD), high-fat diet (HFD), high-fat diet + antioxidant treatment (HFD+Tx), body weight (BW), adiposity index (AI), blood glucose (BG), triglycerides (TG), cholesterol (CL), leptin (LP), heart weight/body weight (HW/BW), blood pressure (BP). Data are the mean ± SEM. n = 10 per group. p<0.01 * SD *vs* HFD, ** HFD *vs* HFD+Tx.

### Effect of Acetylcholine on Coronary Vascular Reactivity

**Fig 1:**
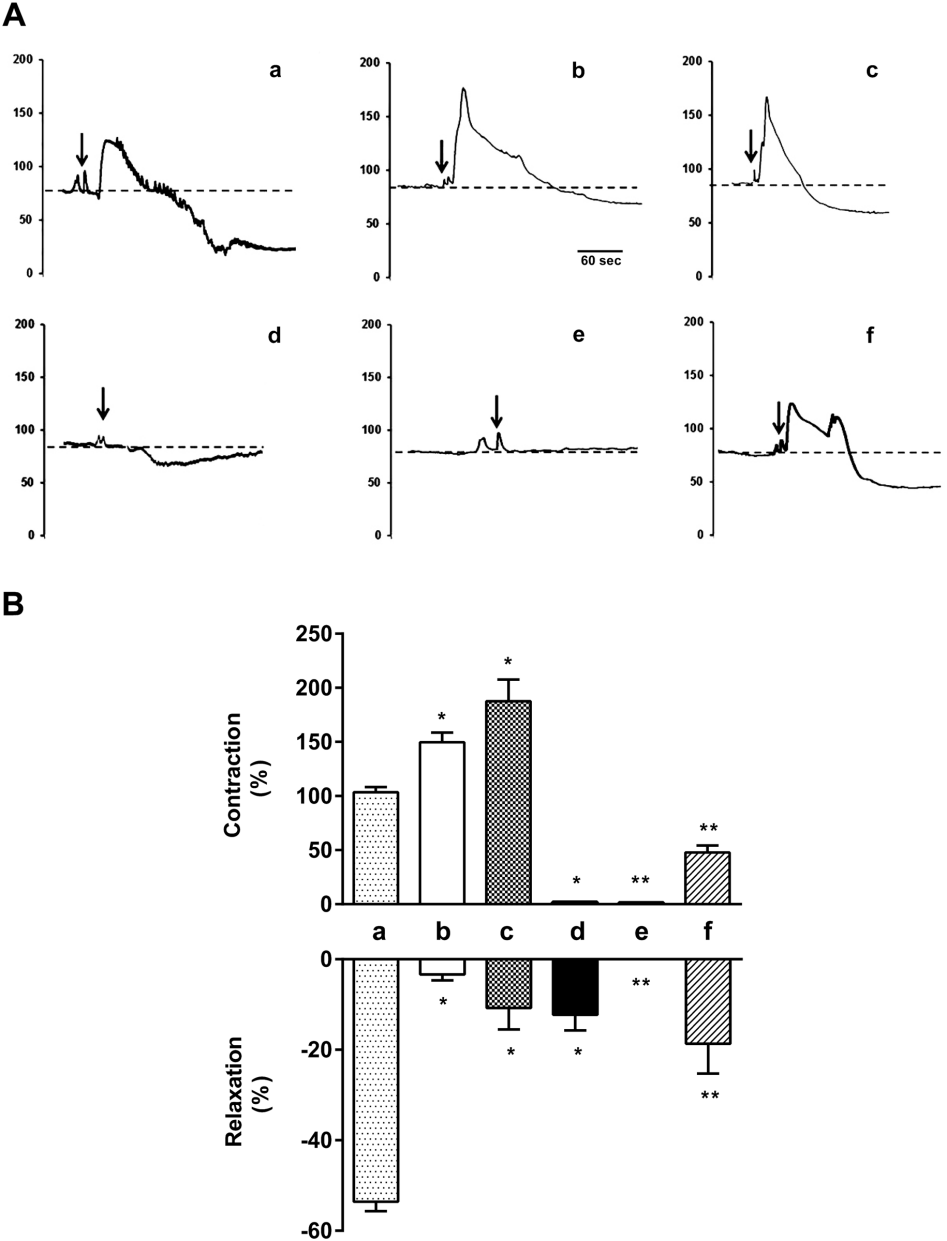
ACh-dependent biphasic effect on coronary pressure in isolated perfused hearts from obese mice. (A) Traces showing a typical ACh biphasic effect of constriction followed by relaxation in coronary circulation. (B) Graphs show the percent of coronary contraction response (top) and relaxation response (bottom) with respect to basal coronary perfusion pressure after ACh bolus administration. Arrows indicate the moment of ACh injection. SD (a), HFD (b), SD+L-NAME (c), SD+indomethacin (d), HFD+indomethacin (e), HFD+Tx (f). n = 10 independent experiments. Data are the mean ± SEM. p<0.05 * vs. SD, ** vs. HFD.

CPP in response to ACh was used to evaluate the effect of diet-induced obesity on coronary vascular reactivity in hearts from SD and HFD mice. Baseline CPP was similar in both groups, 68±8 mmHg in SD mice *vs*. 70±10 mmHg in HFD mice (Fig 1A). In SD coronary arteries, ACh evoked a biphasic response characterized by initial 105±5% increase in CPP (vasoconstriction), compared with baseline pressure value. After reaching the peak, CPP decreased 54±2% below initial baseline pressure values (vasorelaxation) (Fig 1Ba). However in HFD mice, ACh administration induced a marked vasoconstriction phase (157±11% increase in CPP), and a reduced relaxation phase (3.7±1% decrease in CPP) (Fig 1Bb). In the presence of the NOS inhibitor, L-NAME, ACh evoked a greater constriction (187±44% increase in CPP) whereas vasodilatory response was importantly inhibited (10.7±4% decrease in CPP) compared with values in absence of L-NAME in SD mice (Fig 1Bc). In the presence of the non-selective cyclooxygenase inhibitor, indomethacin, ACh-dependent contraction response was totally prevented in SD and HFD mice, while ACh-dependent vasodilatory response was partially reduced (12.3±5% decrease in CPP) in SD and totally blunted in HFD mice, compared with values in absence of indomethacin (Fig 1Bd and 1Be, respectively). The endothelium-independent vasodilatory response to sodium nitroprusside (SNP, 0.01M) was equal in coronary arteries from SD mice and HFD mice, 90% of relaxation. Vasoconstrictor response to the α_1_ adrenergic stimulus was similar in the three experimental groups, thus phenylephrine (1μM)-induced coronary vasoconstrictor responses were 66.9 ±1.5, 68.2 ± 2.0 and 65.7 ±1.8 mmHg in SD, HFD, and HFD treated mice, respectively.

### NO Production in Coronary Arteries

**Fig 2:**
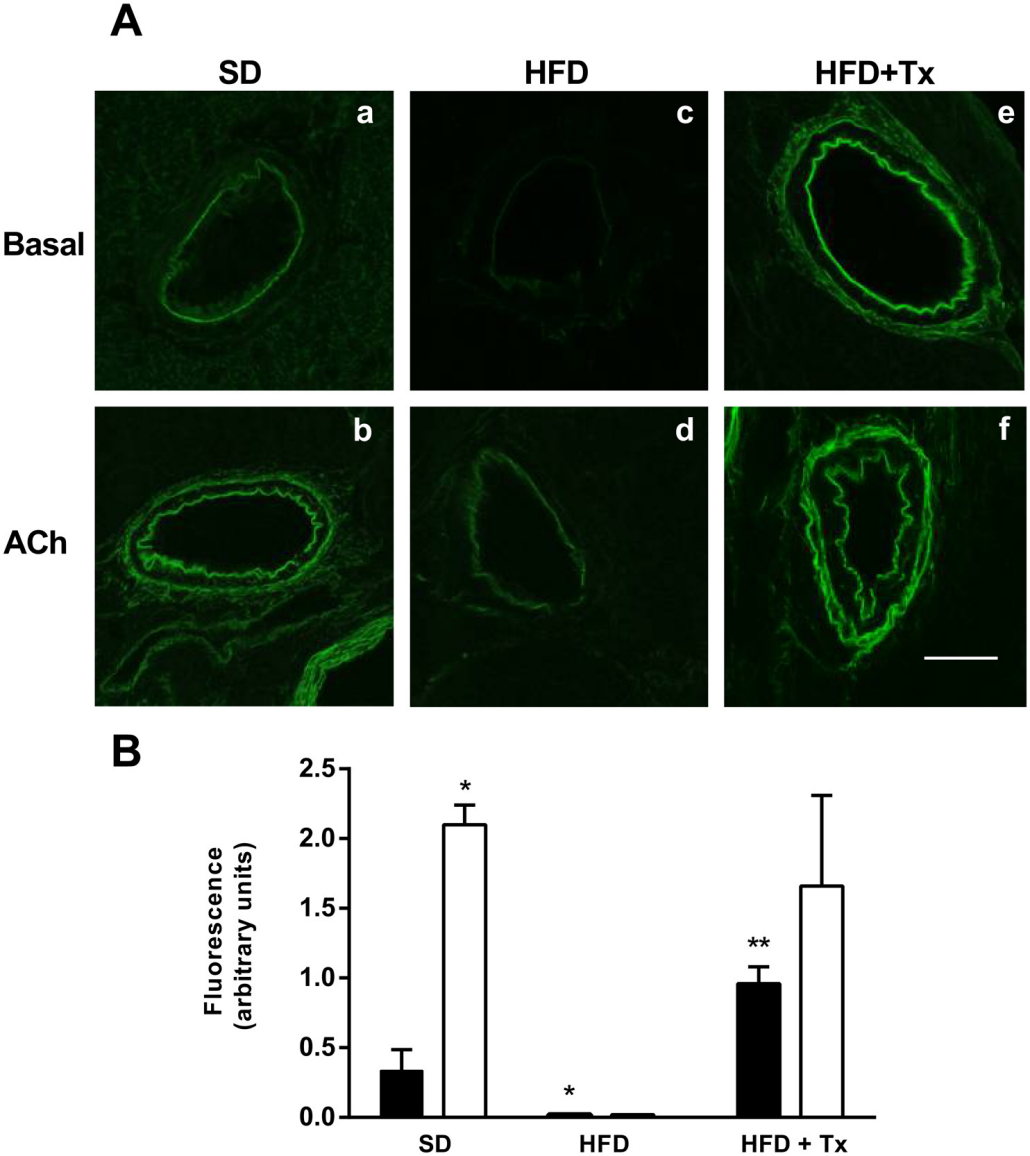
Nitric Oxide (NO) detection in coronary arteries of C57BL/6 mice. (A) Vascular NO depicted by green fluorescence in arteries from standard diet (SD), high-fat diet (HFD), and HFD + antioxidant treatment (HFD+Tx) mice, in basal condition (a, c, e) and after ACh stimulation (b, d, f). Images were captured under identical settings. Magnification 400x. (B) Fluorescence quantification was analyzed by ImageJ software; results are shown in arbitrary units. n = 5 mice per group. Data are the mean ± SEM. p<0.01 * vs SD, ** vs HFD.

To support the functional studies, we determined ACh-induced NO production in the left anterior descending (LAD) and the right coronary (RCA) arteries by using the fluorescent NO indicator DAF2-DA (Fig 2A). The presence of NO depicted as green fluorescence was quantified (Fig 2B) in the coronary arteries from SD hearts in basal conditions (Fig 2Aa), this fluorescence was clearly increased after ACh stimulation (Fig 2Ab) while in coronary arteries from HFD mice, fluorescence was diminished in both, basal and ACh-stimulated conditions (Fig 2Ac and 2Ad, respectively).

### Coronary Artery Prostacyclin and Thromboxane Release

**Fig 3:**
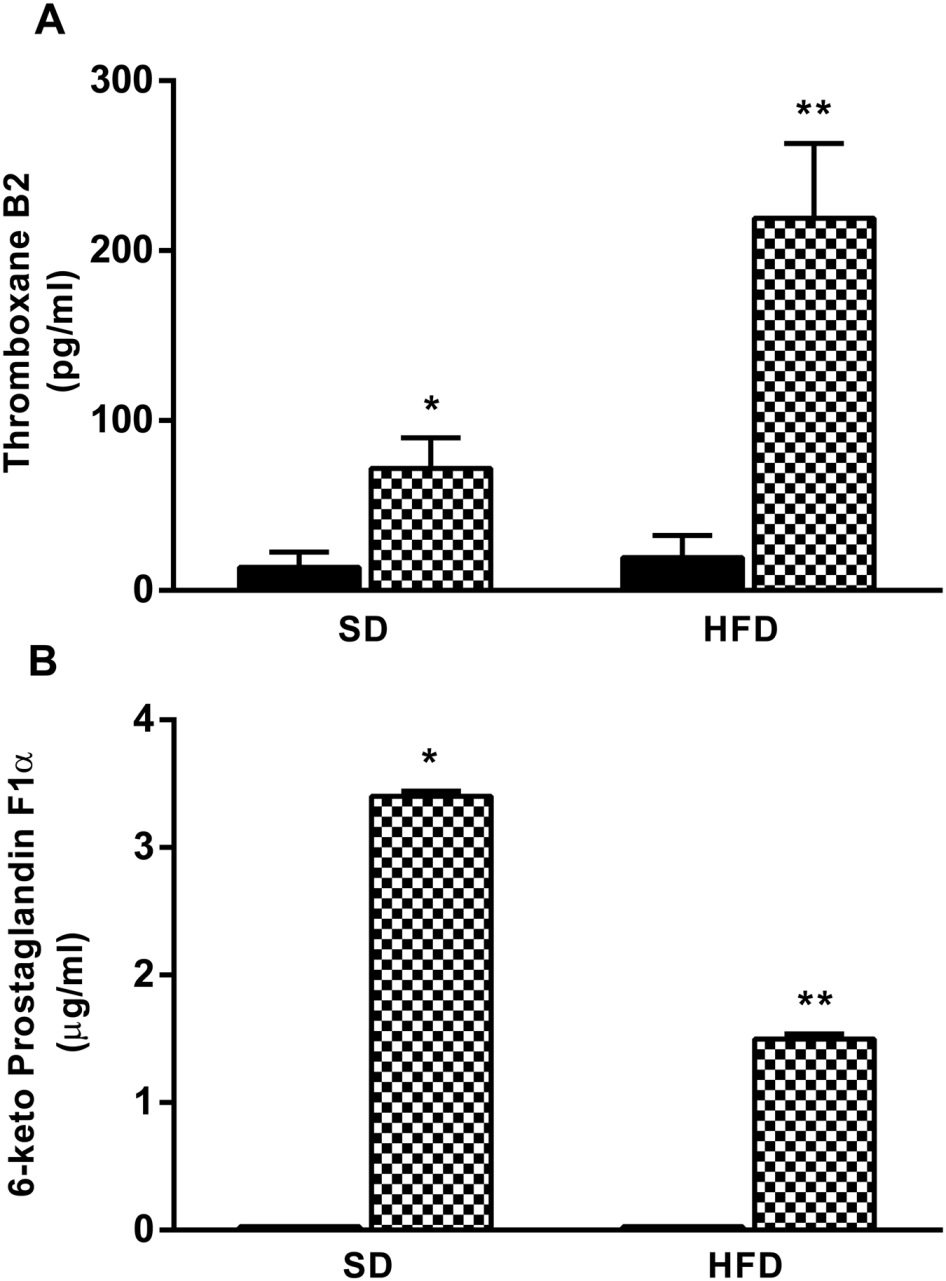
TXA2 and PGI2 release in coronary arteries of obese mice. (A) TXA2 and (B) PGI2 were measured in coronary arteries perfusate from standard diet (SD), and high-fat diet (HFD) mice in basal condition (black bars) or after ACh bolus administration (checkered bars). Perfusates were collected during contraction and relaxation phases. Data are the mean ± SEM of 5 experiments. p<0.05 * vs SD in basal condition, ** vs SD after ACh stimulation.

Perfusates from SD and HFD hearts in basal condition showed similar concentrations of both 6-keto-PGF_1α_ and TXB_2_, the stable metabolites of prostacyclin and TXA_2_, respectively. However, coronary TXA_2_ release during the ACh-induced vasoconstriction phase was increased in HFD compared with SD perfusate after ACh-stimulation (Fig 3A). On the contrary, coronary PGI_2_ release during the ACh-induced vasodilatory response was reduced by half in HFD compared with SD perfusate in the same condition (Fig 3B).

### Coronary Artery Superoxide and Peroxynitrite Production

**Fig 4:**
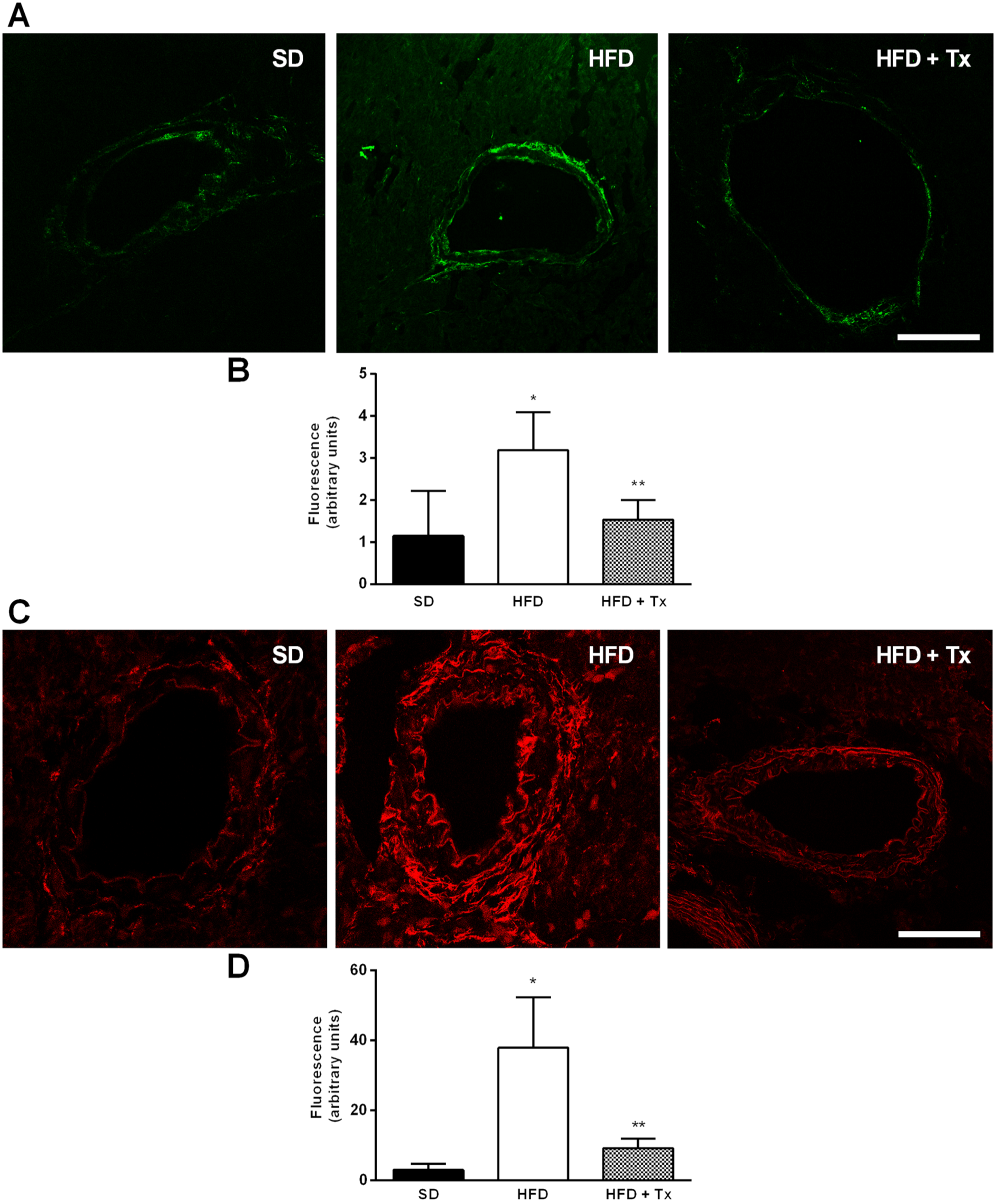
Peroxynitrite and superoxide detection in coronary arteries of obese mice. (A) Vascular peroxynitrite was detected as green fluorescence whereas the presence of (C) vascular superoxide was detected as red fluorescence in unfixed frozen heart sections from standard diet (SD), high-fat diet (HFD), and HFD+ antioxidant treatment (HFD+Tx) mice. Images were captured under identical settings. Magnification 400x. Fluorescence quantification of (B) peroxynitrite and (D) superoxide was analyzed by ImageJ software; results are shown in arbitrary units. n = 5 mice per group. Data are the mean ± SEM. p<0.01 * vs. SD, ** vs. HFD.

Peroxynitrite (Fig 4A) and superoxide (Fig 4C) were detected by the use of specific fluorescence markers. Fluorescence quantification (Fig 4B and 4D) showed low amount of both peroxynitrite and superoxide in coronary arteries from of SD hearts in basal condition (Fig 4A and 4C, SD). However, coronary vascular tissue from HFD mice showed increased fluorescence for both reactive species compared with SD mice, (Fig 4A and 4B, HFD).

### Uncoupled eNOS and Biopterins Determination

**Fig 5:**
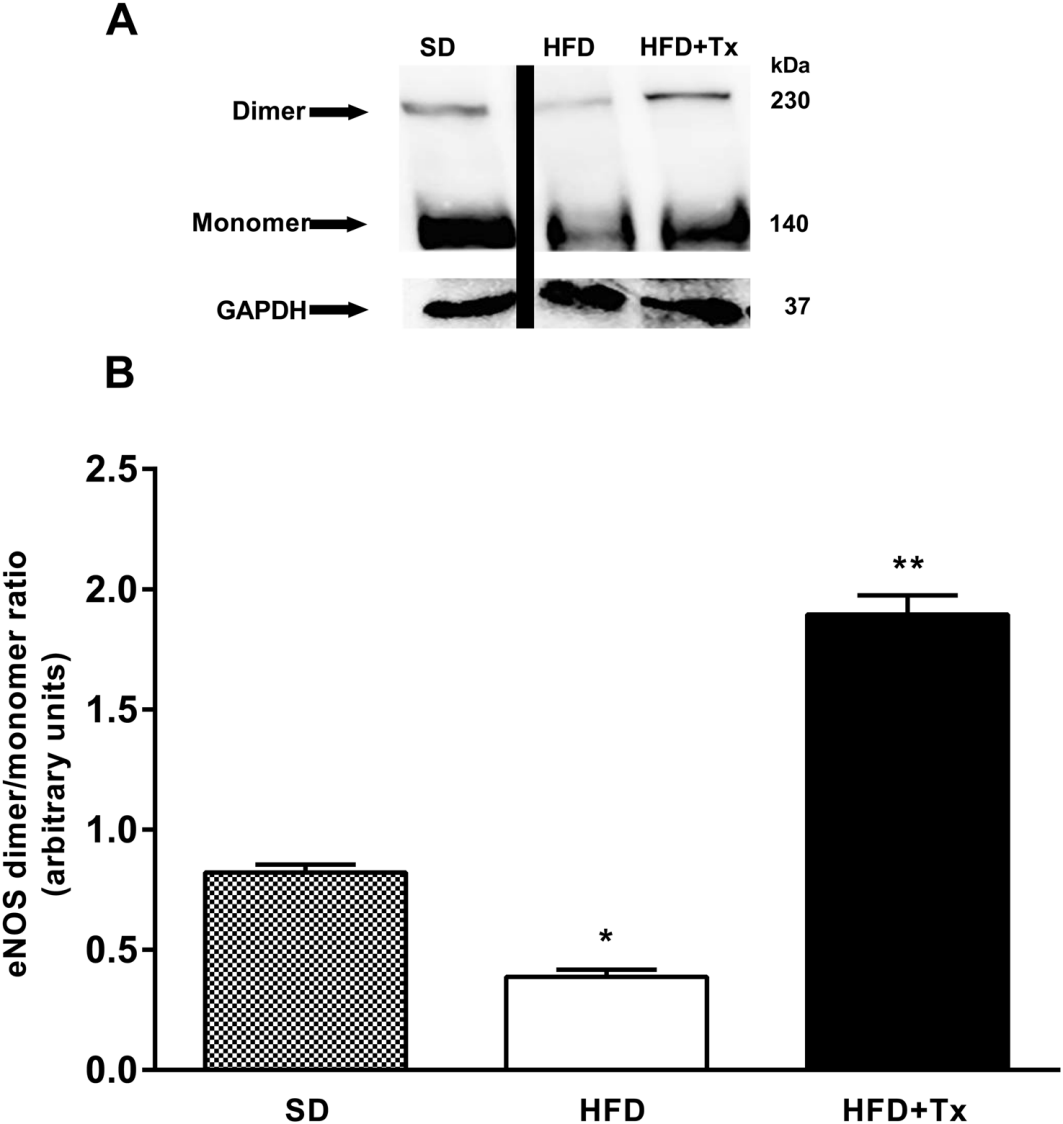
Uncoupled eNOS protein expression in coronary arteries of obese mice. (A) Dimer and monomer protein expression assayed by Western blot with low temperature SDS-PAGE in heart homogenates from standard diet (SD), high-fat diet (HFD), and HFD+ antioxidant treatment (HFD+Tx). (B) Densitometric analysis of the dimer/monomer ratio. n = 5 mice per group. Data are the mean ± SEM. p<0.01* vs. SD, ** vs. HFD.

**Fig 6:**
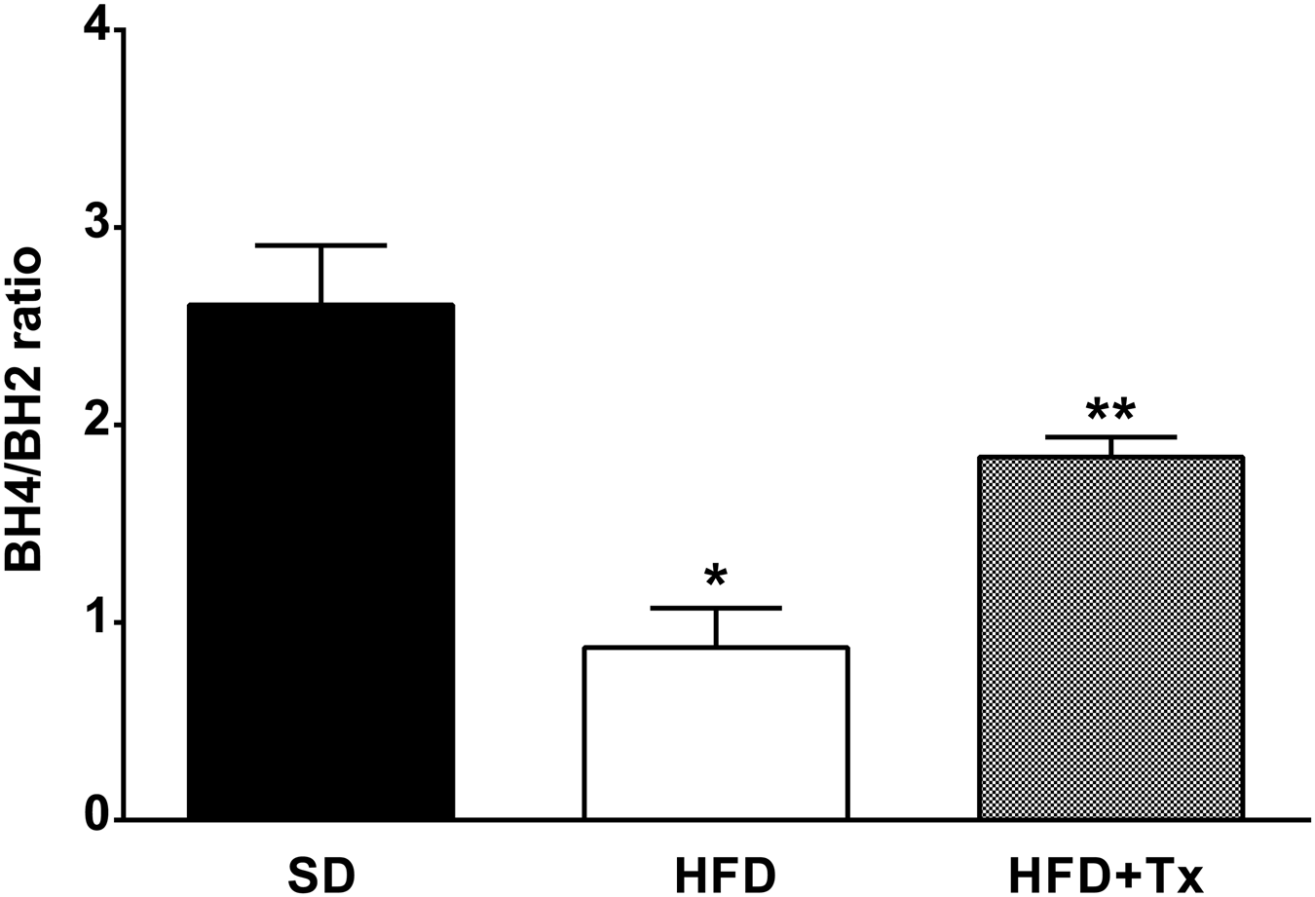
Obesity effect on plasma biopterins concentration. Tetra (BH4) and dihydro (BH2) biopterins concentration was measured in plasma from standard diet (SD), high-fat diet (HFD), and HFD + antioxidant treatment (HFD+Tx) mice and expressed as ratio. n = 5 mice per group. Data are the mean ± SEM. p<0.005 * vs. SD, ** vs. HFD.

Heart tissue from HFD mice expressed reduced eNOS dimer protein expression and similar monomer protein expression when compared with SD mice (Fig 5A). Furthermore, eNOS dimer/monomer ratio was reduced in the HFD mice compared with SD mice (Fig 5B). In the same manner, measurement of plasma biopterins showed that BH4/BH2 ratio was reduced in HFD mice compared with SD mice (Fig 6).

### Antioxidant Treatment Effect on Vascular Reactivity

Chronic treatment with apocynin as antioxidant prevented changes in almost all obesity-related parameters (Table 1). Exceptions were cholesterol and HW/BW ratio. Furthermore, SD treated with the antioxidant apocynin did not alter any of the parameters described in Table 1 (data not shown).

Antioxidant treatment partially restored coronary vascular impairment; it reduced ACh-induced constriction, and improved ACh-dependent vasodilatory response in HFD+Tx mice compared with HFD mice without treatment (Fig 1Bf). Indeed, *in situ* NO levels were higher in both basal and ACh-induced conditions in HFD+Tx mice (Fig 2Ae and 2Af, respectively) compared with HFD mice without treatment (Fig 2Ac and 2Ad, respectively). Vascular generation of peroxynitrite and superoxide was prevented by the antioxidant treatment (Fig 4A and 4C, respectively). Also, eNOS protein expression observed as dimer/monomer ratio (Fig 5B) and plasma BH4/BH2 ratio (Fig 6) were increased 6-fold and 2-fold, respectively, in HFD+Tx mice.

## Discussion

In this study we demonstrated the association between the consumption of a HFD and increased production of ROS/RNS that in turn becomes responsible of the increased synthesis of vascular constrictive factors (TXA_2_) and the diminished release of relaxing substances (NO and PGI_2_), leading to endothelial dysfunction in coronary arteries. Importantly, we have shown that an antioxidant treatment was able to prevent alterations in NO metabolism and thus improved coronary vascular response in obese animals.

The consumption of a HFD during 8 weeks produced a suitable obesity animal model. Thus, we had obese animals resembling human obesity, which is rarely linked to a single gene mutation and can be reversed by restricted caloric intake [16] that allowed us to explore mechanisms associated with cardiovascular diseases during development of obesity. Similar to previous reports [17], the association between a HDF and heart failure was not evidenced in this study. Therefore, we explored coronary vascular function without interference of cardiac dysfunction associated with heart failure.

In this work, ACh elicited a biphasic response in SD mice, characterized by coronary vasoconstriction followed by coronary vasodilation, similar to the response observed in Phe pre-contracted rat renal arteries [18]. However in obese mice, we observed increased vasoconstriction response followed by an importantly reduced vasodilation phase, suggesting that obesity impairs ACh-induced vasodilation. Indeed, obesity is an independent risk factor for endothelial dysfunction [19, 20]. Experimental evidence supports that reduced NO bioavailability resulted in a reduced agonist-induced dilation of mesenteric and skeletal muscle microvessels of obese mice and rats [21, 22]. In accordance with our results, previous studies have shown that impaired ACh-induced vasodilation in the microvasculature was associated with decreased NO expression in hamsters on a HDF for 12 weeks [23]. Also, studies in humans have demonstrated reduced endothelium-dependent vasodilation in obese subjects [24, 25]. As the presence of a biphasic response in coronary arteries from SD mice suggests a functional competition between relaxing and contractile mechanisms [26], reduced vasodilation in HFD mice may be related either with reduced vasorelaxing factors, increased vasoconstrictor mechanisms, or both. Indeed, several authors have demonstrated increased vasoconstriction in cardiovascular diseases that included obese and hypertensive animal models [27, 28, 29]. Moreover, increased coronary resistance during obesity has been reported recently [30]. These authors suggested that decreased coronary blood flow in obesity was related to changes in heart contraction rather than changes in coronary vascular mechanisms as suggested by our study. Additionally, impaired vasodilation in response to cholinergic stimulation in hypertensive patients has been demonstrated, suggesting impairment on NO as one of the responsible mechanisms [31]. However, it is not well known whether endothelial dysfunction is cause or consequence of hypertension and in our study, cardiac hypertrophy was not evident regardless of hypertension, as demonstrated by heart weight/body weight and heart weight/femur length ratios in obese mice.

Since the discovery of endothelium-derived relaxing factor by Furchgott and Zawdazki (1980), ACh-induced vasodilation has been widely studied as indication of NO-dependent endothelium response [32]. In this work, NO-dependent ACh vasodilation in SD mice was evidenced by inhibition of eNOS by L-NAME, thus decreased ACh vasodilation in obese mice can be related with loss of NO synthesis. This idea was supported by the decreased NO levels found in HFD coronary arteries in both basal condition and after ACh intracoronary injection compared with SD mice.

Furthermore, the NO-independent, ACh-evoked coronary vasodilation demonstrated here can be mediated via PGs pathway besides NO as suggested by other studies [33, 34, 35]. Accordingly, our data showed diminished ACh-dependent coronary vasodilation in the presence of cyclooxygenase inhibition together with decreased PGI_2_ levels in obese mice. This result suggests that both NO and PGI_2_ reduced bioavailability contributed to the development of endothelial dysfunction in coronary arteries from obese mice. Hodnett et al. (2009) found diminished release of arachidonic acid-induced PGI_2_ in femoral arteries from obese Zucker rats [36].

In addition, ACh-induced, TXA_2_ mediated vasoconstriction has been previously observed in rabbit pulmonary arteries [37]. Thus, impaired relaxation response in obese mice could be related with increased production of the vasoconstrictive metabolite and might limit vasorelaxation in response to other mechanisms. The ACh-mediated contraction response was abolished by indomethacin suggesting an arachidonic acid product as endothelial contracting factor. Indeed, the increased concentration of TXA_2_ found in perfusates collected during contraction phase in obese animals supports this idea. Accordingly, enhanced TXA_2_ production has been demonstrated in several cardiovascular diseases like unstable angina, acute myocardial infarction [38], spontaneous hypertension [39], and hypercholesterolemia [40]. Also, increased TXA_2_ synthesis is supported by previous reports of highly expressed COX_2_ in obese rats [41]. Moreover, changes in thromboxane receptor (TP) expression were not involved in the enhanced TXA_2_ response observed, since the contractile response to TP agonist U46619 (9,11-dideoxy-9α,11α-methanoepoxy PGF_2α_) was similar in SD and HFD mice (data not shown).

The association obesity-endothelial dysfunction involves several mechanisms that include increased plasma leptin levels. The adipocyte-derived hormone leptin has been shown as modulator of vascular tone and NO production [42]. Long time exposure of endothelial cells to leptin resulted in diminished NO bioavailability [43]. NO degradation by ROS has been suggested as the mechanism involved in this leptin decreased NO-dependent vasodilation [44]. Additionaly, leptin has been associated with proinflammatory and immune stimulatory mechanisms along with ROS formation in obese mice, suggesting these processes as the responsible for the leptin atherogenic effect that leads to endothelial dysfunction [45]. In the present study, we neither observed lipid nor endothelial lesions in the coronary vessels of obese mice after 8 weeks on a HFD, suggesting that leptin-dependent endothelial injury is not associated with impaired coronary relaxation. The hyperleptinemia observed in HFD mice supports this idea and may be partially responsible for the endothelial dysfunction, although the precise mechanisms are not fully understood.

It has been widely reported that oxidative stress is implicated in a variety of physiological and pathological processes that include aging, cancer, diabetes, and atherosclerosis [46], and plays an important role in the development of endothelial dysfunction and vascular diseases [20, 47, 48]. Hence, an important possible mechanism associated with the decreased NO and PGI_2_ bioavailability in obese mice observed in this study might be the presence of increased ROS/RNS in vascular tissue, as suggested by a previous study from our laboratory [10]. In the same manner, endothelium-dependent contractions were abolished by superoxide dismutase in canine basilar artery [49]. Also, other studies have demonstrated that PGI_2_ synthase is inactivated by peroxynitrite [50, 51]. All these findings support our hypothesis of impairment of PGI_2_ synthesis during ACh-dependent relaxation response in obese animals, however further experiments are needed to explain this possible mechanism.

The idea of oxidative stress being responsible for the changes in ACh-induced coronary response is supported by our data that showed enhanced superoxide and peroxynitrite production in the coronary arteries from obese mice. NO and superoxide react rapidly to form peroxynitrite and previous reports have shown in the vasculature that peroxynitrite oxidizes BH4, an essential cofactor for eNOS, to its oxidative form BH2 [52]. As a result, abnormally low levels of BH4 promote eNOS uncoupling with concomitant decrease in NO release and subsequent loss of vasorelaxation [16, 18]. In this study, a low BH4/BH2 ratio was observed in HFD mice.

The proposal of ROS production as the leading mechanism for endothelium dysfunction through reduced NO synthesis is further sustained by concomitant administration of a high-fat diet and an antioxidant treatment that resulted in prevention of obesity development, ACh coronary vasodilation impairment, and avoidance of eNOS uncoupling and NO synthesis. Similarly, ROS-mediated endothelium impairment in coronary arteries from obese and diabetic mice has been demonstrated [53]. Apocynin has been widely used in research as NADPH oxidase inhibitor, a major source of vascular ROS [27, 54, 55]. Consistent with our results, several authors have shown similar apocynin effects on obesity [27, 54]. Also, Hayashi, et al (2005) showed that apocynin treatment reversed endothelial dysfunction in diabetic rats [56]. Furthermore, antioxidant treatment prevented eNOS uncoupling and improved BH4/BH2 ratio to higher levels than control. Clinical and experimental studies have shown endothelial dysfunction improvement after administration of BH4 in patients with hypercholesterolemia [57], and increased total biopterin and BH4 levels in aorta along with endothelial dysfunction improvement in apoE-deficient mice after antioxidant treatment [58]. Despite these reports along with this study, the exact mechanism by which apocynin prevents those metabolic and biochemical changes is still not clear.

Obesity can be viewed as a state of chronic oxidative stress and this can cause the development of alterations in vascular reactivity. However, the impossibility to differentiate whether the beneficial effect on endothelial function achieved with the apocynin treatment was associated with decreased leptin plasma concentrations or with coronary blood flow changes *in vivo* arises as the limitation of this study. Experiments using in *vivo* leptin administration or ob/ob mice may be needed to further explore leptin role in coronary dysfunction in obese mice. Also, we are describing coronary vasodilation impairment in isolated perfused heart system without the normal feedback mechanisms that integrate coronary circulation flow, then it is possible that in living animal this coronary relaxing impairment may be compensated by adrenergic or hormonal influences not appreciated in our system. *In vivo* coronary circulation experiments are required to further confirm our findings.

In conclusion, we demonstrated that obesity causes coronary endothelium dysfunction due to an imbalance in the production of vasorelaxing and vasoconstricting substances, through the increased generation of oxidative stress that in turn affects eNOS and cyclooxygenase activities. Further studies are necessary to elucidate timing and effectiveness in therapies to prevent coronary endothelial dysfunction related with obesity-dependent oxidative stress.
